# Attitudes related to technology for active and healthy aging in a national multigenerational survey

**DOI:** 10.1038/s43587-023-00392-3

**Published:** 2023-04-06

**Authors:** J. Offerman, S. Fristedt, S. M. Schmidt, C. Lofqvist, S. Iwarsson

**Affiliations:** 1 Department of Health Science, Faculty of Medicine, Lund University, Lund, Sweden; 2 Jönköping Academy for Improvement of Health and Welfare, School of Health and Welfare, Jönköping University, Jönköping, Sweden

**Keywords:** Social sciences, Policy, Medical research, Ageing

## Abstract

Research is needed to understand attitudes toward and adoption of the broad range of technologies available to support active and healthy aging in different generations. The present article gives an overview of the GenerationTech survey and sample, and describes attitudes and acceptance related to technology in general and as a means to support active and healthy aging. A national survey was conducted with a random sample (*n* = 2,121) including men and women from three generations (30–39, 50–59 and 70–79-year-olds) in Sweden. The generations shared some attitudes toward and acceptance of technologies for active and healthy aging. However, what kind of technologies are preferred to support active and healthy aging and the reasons for using certain technologies differed by generation. The findings could help guide the development and implementation of technologies for active and healthy aging throughout the aging process.

## Abstract (web summary)

Technologies could aid active and healthy aging but their development and implementation requires knowledge of user preferences. In this study, the authors conducted a Swedish national survey across three generations to understand attitudes toward technologies.

## Main

The rapid technological development seen today promises new ways and means to support active and healthy aging^1–3^, but the actual capability of technologies to offer such support is underexplored^4^. A definition of active and healthy aging applicable in this context is “the process of optimizing opportunities for health, participation, and security to enhance quality of life as people age”^5^.

Although a broad spectrum of technologies is well integrated in people’s everyday lives and routines, research tends to neglect the existing diversity of products and services. According to a literature review, multiple studies describe attitudes toward and adoption of information and communication technology (ICT), e-health, wearable or home-based health monitoring systems and smart home technology^6^; however, other types of technology used in everyday life, such as kitchen appliances, other household equipment and cars, are scarcely considered. While the positive effects of using technologies for monitoring physical, cognitive and mental health are well known, research on how technology can support active and healthy aging is scarce. The results that exist are conflicting^7^ and better-quality evidence is needed^8^. Similarly, there is a need for larger quantitative studies to better understand the relationships between factors that influence said acceptance^9,10^.

According to the unified theory of acceptance and use of technology (UTAUT), adoption of technology is explained by taking user characteristics (age, sex and experience) into consideration, Moreover, in UTAUT, three factors of intention to use (performance expectancy, effort expectancy and social influence) and two determining factors of usage behavior (intention and facilitating conditions) are used to explain the adoption of technology^11^. The successor UTAUT2 incorporates three new constructs: hedonic motivation; price and value; and habit. These two commonly used models (UTAUT and UTAUT2) have been shown to be relevant for explaining the adoption and acceptance of new technologies and reflects the end user characteristics^12,13^.

However, both UTAUT and UTAUT2 focus on user’s adoption of technology rather than adapting technology to the user’s needs. Thus, more research is urgently needed to explore the adoption of technology and how technology can be better adapted to the needs of end users, not only among current older populations but also throughout the course of aging. In fact, few studies have a longitudinal design or have been designed to identify generation and cohort effects^14,15^. Studies contemplating the differences among^16^, within or across different age groups are especially lacking. Although studies have shown that there are age disparities when it comes to attitudes and confidence toward technology, older adults have a more negative attitude toward technology and feel less comfort and efficacy about using computers^17^. Belonging to a different technological generation may explain why groups of older adults experience problems with current ICT products^16,18^. However, the relationship between older people’s technological needs and their previous knowledge and experience is largely unexplored^3,18^. Early use of technology is a strong predictor for later use of technology and use during childhood and adolescence lays the foundation for an individual’s experience with technology^19^.

Previous research has mainly focused on the adoption of technology by older adults and their difficulties with technology, presenting them as technophobic and having lower levels of computer literacy^20^. Findings from a meta-analysis challenged such stereotypes as technology acceptance in older adults showed a negative relationship with chronological age only for technologies that had no clear perceived functionality (for example, social media) in the lives of older adults^15^. According to Berkowsky et al.^21^ adults are more likely to adopt technology if they perceive it is of value to them and if it will positively impact their lives. Moreover, perceived usefulness and perceived ease of use facilitated acceptance and demonstrated a positive correlation with older adults’ attitudes toward technology; older adults attach importance to the benefits expected from the use of technology products^22^.

Addressing the paucity of research focused on the perceptions of and attitudes toward technology adoption and usage among people of different generations as related to the technological developments they have experienced during their lifetime, we undertook the GenerationTech project. The unique perspective is to address aging and technology from a generational perspective as related to health, with specific attention to cohort and period similarities and differences. The project is founded on a combination of theories on health, environmental gerontology, age stratification and technology. Using quantitative and qualitative data, the ambitious mixed methods design rests on an interdisciplinary and transdisciplinary approach.

Challenging ageistic stereotypes, the first study from GenerationTech^23^ found that attitudes to technology were individually minded rather than generational as people from three generations shared perspectives across rather than within generations. The current quantitative study is a first contribution, to our knowledge, in terms of more generalizable knowledge. These two studies are both part of GenerationTech, designed to address urgent knowledge gaps with regard to the complex interaction between generations of the aging population and their adoption of a wide range of technologies, and how this has a role for active and healthy aging (Table 1).

**Table 1:** Sampling frame, number of respondents, response rate and response mode across the three age cohorts (*n* = 2,121)

Age cohort, years	Sampling frame	No. of respondents (response rate (%))	Total no. of responses (response rate (%) online/phone/mail^a^)
	Men/women	Men/women	
30–39	2,300/2,300	316 (14)/323 (14)	639 (97/2/1)
50–59	1,500/1,500	345 (23)/358 (24)	703 (96/3/1)
70–79	1,200/1,200	420 (35)/359 (30)	779 (93/5/2)
Total	5,000/5,000	1,081 (22)/1,040 (21)	2,121 (95/4/1)

^a^Mode of response.

The purpose of this first study from the GenerationTech survey was to give an overview of the sample and survey methodology, followed by a description of attitudes and adoption related to technology in general and as a means to support active and healthy aging from the perspective of three generations. Observing age cohort and sex similarities and differences the following research questions guided the analyses: What were the attitudes to different types of technology and how did these differ among three generations? What products were perceived to be most important for an active and healthy aging process, and were there differences among the generations? What product characteristics did adults in the three generations consider important when adopting or accepting new technology?

## Results

In total, 2,121 people completed the survey including men and women from three different generations (30–39, 50–59 and 70–79 years). Almost all were born in Sweden and had at least a compulsory school level education. The youngest generation had a higher education and rated their general health better than the other generations. The oldest generation rated their economy and general health lower than the other generations. The GenerationTech survey sample in terms of birthplace, education, occupation, self-rated economy, subjective or self-rated health status, birth country and current place of residence is presented in Table 2. Although the internal response rate was generally complete, 138 of respondents refrained from reporting their birthplace.

**Table 2:** Characteristics of the GenerationTech survey sample (*n* = 2,121)

Characteristic	Age 30–39	Age 50–59	Age 70–79
	*n* = 639	*n* = 703	*n* = 779
	% (*n*)	% (*n*)	% (*n*)
**Sex**			
Male	49 (316)	49 (345)	54 (420)
Female	51 (323)	51 (358)	46 (359)
**Country of birth**			
Sweden	88 (533)	89 (569)	93 (684)
Europe	6 (36)	8 (53)	6 (42)
Other	6 (36)	3 (19)	1 (7)
**Education**			
Compulsory school	2 (14)	4 (30)	28 (217)
High school	24 (153)	35 (244)	14 (111)
Polytechnic	14 (85)	12 (83)	17 (130)
University	60 (383)	49 (341)	41 (314)
**Main occupation**			
Studying	5 (29)	1 (6)	<1 (3)
Working	83 (527)	90 (630)	2 (13)
Maternity or paternity leave	7 (43)	0 (0)	0 (0)
Retired	<1 (1)	2 (13)	95 (735)
Unemployed	2 (16)	3 (19)	0 (0)
Other	3 (18)	4 (28)	3 (23)
**Size of municipality (*n*** **inhabitants)**			
>200,000	43 (273)	35 (243)	31 (244)
>40,000	37 (233)	39 (272)	39 (302)
>15,000	16 (106)	21 (144)	21 (160)
Rural municipality	4 (25)	5 (38)	9 (68)
**Subjective economy for technology needs**			
Well	52 (333)	55 (382)	40 (305)
Fairly well	36 (228)	34 (239)	44 (338)
Fairly bad	9 (55)	7 (51)	10 (76)
Bad	3 (21)	4 (25)	6 (52)
**Self-rated general health**			
Excellent	21 (130)	17 (120)	8 (64)
Very Good	41 (264)	39 (275)	32 (242)
Good	29 (182)	31 (213)	39 (302)
Fair	8 (52)	10 (69)	19 (143)
Poor	1 (9)	3 (21)	2 (17)
**Self-rated life satisfaction**			
Excellent	15 (96)	17 (115)	16 (120)
Very Good	46 (290)	44 (307)	39 (301)
Good	29 (183)	28 (193)	34 (258)
Fair	8 (53)	9 (66)	10 (80)
Poor	2 (12)	2 (13)	1 (6)

Numbers are expressed as the percentage of each age cohort and rounded to the nearest integer.

### Attitudes toward technology for active and healthy aging

**Fig. 1:**
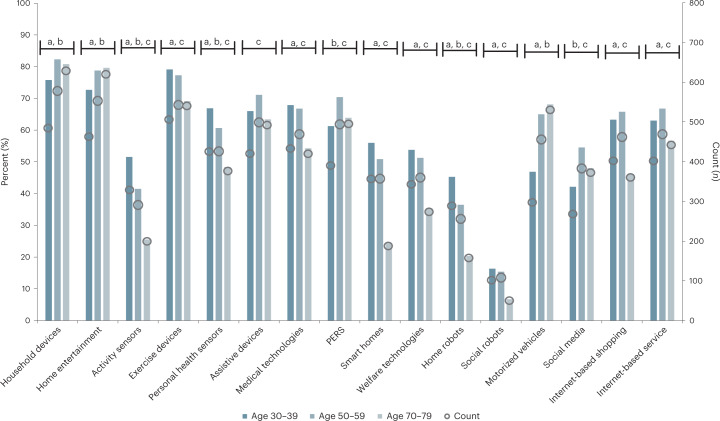
Technologies that respondents liked to use for active and healthy aging (*n* = 2,121). The bars represent each generation’s response to what kind of technology they wanted for active and healthy aging. Percentages were rounded to the nearest integer. The dots represent how many from each generation responded to what reasons they had for using technologies for active and healthy aging. Key to the bars in the bar chart: ‘a’ represents the oldest generation (70–79-year-olds), which differed significantly from the youngest generation (30–39-year-olds) (*P* < 0.05); ‘b’ represents the youngest generation, which differed significantly from the middle-aged generation (*P* < 0.05); and ‘c’ represents the oldest generation, which differed significantly from the middle-aged generation (*P* < 0.05). All pairwise comparisons were analyzed with a two-sided chi-squared test. All *P* values were Bonferroni-corrected.

To support active and healthy aging, respondents preferred using household devices, home entertainment, exercise devices and assistive devices. The oldest (70–79 years) generation compared to the other generations was significantly less interested in using activity sensors, exercise devices, personal health sensors, medical technologies, smart homes, welfare technologies, home and social robots, Internet shopping and Internet services to support active and healthy aging. The youngest (30–39 years) generation compared to the oldest generation was significantly less interested in using household devices, home entertainment, motorized vehicles and social media to support active and healthy aging. The middle-aged (50–59 years) generation compared to the oldest generation was significantly more interested in using assistive devices, personal emergency response systems (PERS) and social media to support active and healthy aging (Fig. 1). All pairwise comparisons are presented in Extended Data Table 1.

**Fig. 2:**
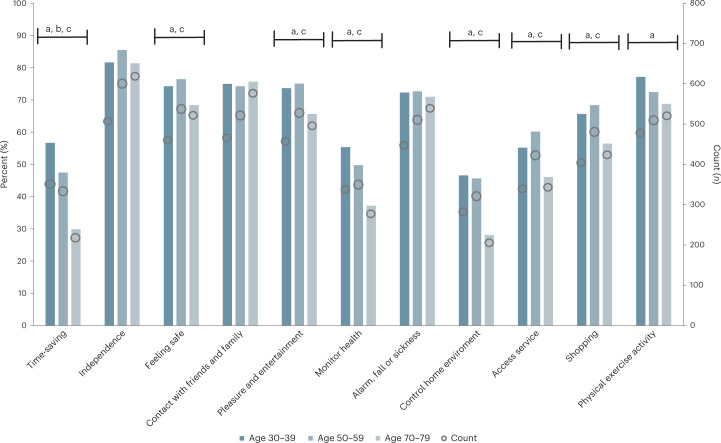
Respondents’ reasons for using technologies to promote active and healthy aging (*n* = 2,121). The bars represent each generation’s response to what reasons they had for using technologies for active and healthy aging. Percentages were rounded to the nearest integer. The dots represent how many from each generation responded to what reasons they had for using technologies for active and healthy aging. Key to the bars in the bar chart: ‘a’ represents the oldest generation (70–79-year-olds), which differed significantly from the youngest generation (30–39-year-olds) (*P* < 0.05); ‘b’ represents the youngest generation, which differed significantly from the middle-aged generation (*P* < 0.05); and ‘c’, represents the oldest generation, which differed significantly from the middle-aged generation (*P* < 0.05). All pairwise comparisons were analyzed with a two-sided chi-squared test. All *P* values were Bonferroni-corrected.

The primary reasons reported for wanting to use technologies were to be independent, remain in contact with friends and family, be physically active and notify someone in case of a fall or illness. The oldest generation compared to the other generations was significantly less interested in using technologies to save time, feel safe, monitor health, control home entertainment, access services, for pleasure and entertainment or shopping. The middle-aged generation compared to the youngest generation was significantly less interested in using technologies to save time (Fig. 2). All pairwise comparisons are presented in Extended Data Table 2.

Overall, respondents considered household devices to be practical and necessary to meet their needs. However, the oldest generation had a significantly lower odds ratio (OR) of perceiving household devices as useful (OR = 0.58, 95% confidence interval (CI) = 0.47–0.72), user-friendly (OR = 0.81, 95% CI = 0.65–0.99) and time-saving (OR = 0.80, 95% CI = 0.64–1) compared to the youngest generation, and a significantly higher OR to acknowledge that household devices brought them independence (OR = 1.73, 95% CI = 1.34–2.23) compared to the youngest generation. The middle-aged generation had a significantly lower OR of perceiving household devices as user-friendly (OR = 0.81, 95% CI = 0.65–0.99) and a significantly higher OR of perceiving household devices as time-saving (OR = 1.29, 95% CI = 1.01–1.63) and acknowledging that such products brought them independence (OR = 1.5, 95% CI = 1.18–2) compared to the youngest generation. Results from all binary logistic regressions are presented in Table 3. Taking socio-demographic characteristics into consideration, we noticed in the adjusted model that socio-demographic factors had a minor moderating effect on some of the attitudes (for example, time-saving and make me independent) (Table 3).

**Table 3:** Respondents’ attitudes toward household devices based on binary logistic regression, presenting unadjusted and adjusted models controlling for confounding variables (*n* = 2,121)

	Percentage	Unadjusted model	Adjusted model
Attitudes	*n* (%)	OR (95% CI)	OR (95% CI)
**Useful**			
30–39 years	65	Ref.	Ref.
50–59 years	65	1.00 (0.80–1.25)	0.99 (0.77–1.25)
70–79 years	52	0.58 (0.47–0.72)	0.58 (0.46–0.74)
**User-friendly**			
30–39 years	48	Ref.	Ref.
50–59 years	43	0.81 (0.65–0.99)	0.78 (0.62–0.98)
70–79 years	35	0.59 (0.48–0.73)	0.61 (0.48–0.77)
**Necessary need**			
30–39 years	68	Ref.	Ref.
50–59 years	72	1.23 (0.97–1.56)	1.24 (0.96–1.59)
70–79 years	69	1.05 (0.84–1.32)	1.13 (0.88–1.46)
**Practical**			
30–39 years	71	Ref.	Ref.
50–59 years	72	1.01 (0.80–1.28)	1.08 (0.84–1.39)
70–79 years	67	0.80 (0.64–1.01)	0.94 (0.73–1.22)
**Time-saving**			
30–39 years	69	Ref.	Ref.
50–59 years	74	1.29 (1.01–1.63)	1.33 (1.03–1.72)
70–79 years	64	0.80 (0.64–1.00)	0.96 (0.75–1.24)
**Trustworthy**			
30–39 years	34	Ref.	Ref.
50–59 years	36	1.11 (0.88–1.38)	1.08 (0.85–1.38)
70–79 years	32	0.94 (0.75–1.17)	1.03 (0.80–1.33)
**Safe to use**			
30–39 years	44	Ref.	Ref.
50–59 years	46	1.12 (0.86–1.36)	1.16 (0.92–1.47)
70–79 years	40	0.87 (0.70–1.07)	0.95 (0.74–1.20)
**Make me independent**			
30–39 years	18	Ref.	Ref.
50–59 years	25	1.51 (1.18–2.00)	1.62 (1.22–2.14)
70–79 years	28	1.73 (1.34–2.23)	1.85 (1.39–2.64)

The dependent variable is the attitude toward household devices and the independent variables are the generation one belongs to and confounding variables. Ref., reference.

Most of the respondents perceived ICTs to be useful, practical, time-saving and meeting their necessary needs. However, the oldest generation had a significantly lower OR of perceiving ICT products as useful (OR = 0.31, 95% CI = 0.25–0.39), user-friendly (OR = 0.31, 95% CI = 0.25–0.39), necessary need (OR = 0.71, 95% CI = 0.62–0.95), practical (OR = 0.76, 95% CI = 0.60–0.95) and time-saving (OR = 0.58, 95% CI = 0.47–0.72) compared to the youngest generation; 50–59-year-olds had a significantly lower OR of perceiving ICT products as useful (OR = 0.74, 95% CI = 0.58–0.93), user-friendly (OR = 0.53, 95% CI = 0.43–0.66) and being practical (OR = 0.76, 95% CI = 0.60–0.95) compared to the youngest generation. Middle-aged individuals had a significantly higher OR of acknowledging that ICT brought them independence (OR = 1.43, 95% CI = 1.14–1.8) compared to the youngest generation. Results from all binary logistic regressions are presented in Table 4. Taking socio-demographic characteristics into consideration, we noticed in the adjusted model that socio-demographic factors had a minor moderating effect on some of the attitudes (for example, time-saving and make me independent) (Table 4).

**Table 4:** Respondents’ attitudes toward ICT products based on logistic regression, unadjusted and adjusted models controlling for confounding variables (*n* = 2,121)

	Percentage	Unadjusted model	Adjusted model
Attitudes	*n* (%)	OR (95% CI)	OR (95% CI)
**Useful**			
30–39 years	73	Ref.	Ref.
50–59 years	67	0.74 (0.58–0.93)	0.72 (0.56–0.93)
70–79 years	57	0.49 (0.39–0.61)	0.54 (0.42–0.70)
**User-friendly**			
30–39 years	50	Ref.	Ref.
50–59 years	35	0.53 (0.43–0.66)	0.52 (0.41–0.66)
70–79 years	24	0.31 (0.25–0.39)	0.33 (0.25–0.43)
**Necessary need**			
30–39 years	59	Ref.	Ref.
50–59 years	61	1.08 (0.86–1.34)	1.08 (0.86–1.36)
70–79 years	53	0.77 (0.62–0.95)	0.84 (0.67–1.07)
**Practical**			
30–39 years	70	Ref.	Ref.
50–59 years	64	0.76 (0.60–0.95)	0.81 (0.63–1.03)
70–79 years	50	0.43 (0.34–0.53)	0.46 (0.36–0.59)
**Time-saving**			
30–39 years	61	Ref.	Ref.
50–59 years	60	0.98 (0.79–1.22)	1.05 (0.83–1.33)
70–79 years	47	0.58 (0.47–0.72)	0.73 (0.57–0.93)
**Trustworthy**			
30–39 years	20	Ref.	Ref.
50–59 years	20	0.99 (0.75–1.29)	1.03 (0.78–1.38)
70–79 years	16	0.75 (0.57–1.01)	0.84 (0.62–1.14)
**Safe to use**			
30–39 years	22	Ref.	Ref.
50–59 years	22	0.98 (0.76–1.28)	1.02 (0.78–1.35)
70–79 years	19	0.82 (0.63–1.07)	0.92 (0.69–1.35)
**Make me independent**			
30–39 years	30	Ref.	Ref.
50–59 years	38	1.43 (1.14–1.80)	1.45 (1.13–1.85)
70–79 years	33	1.15 (0.92–1.45)	1.39 (1.08–1.79)

The dependent variable is the attitude toward ICT and the independent variables are the generation one belongs to and confounding variables.

#### Important factors when choosing and adopting technology

The responses show that price, technology allowing flexible use and standard rather than extra functions matter when choosing new products. Overall, respondents reported that they learnt new products easily and had no problems keeping up with technology development. The oldest generation especially considered environmental sustainability important when adopting new technologies. Always wanting the latest was not considered an important factor when adopting new technologies for most respondents. Respondents’ answers to important factors when choosing and adopting technology are presented in Extended Data Tables 3 and 4.

## Discussion

Going beyond perspectives from single age groups in later life, the present study reports similarities and differences across generations rarely displayed in research on aging and technology. Across the three targeted generations, respondents preferred traditional technologies (that is, household devices, assistive devices) rather than more recently introduced technologies (smart homes and welfare technologies) to support their active and healthy aging. Moreover, we found variations in attitudes toward technology and reasons for using technology between generations. Additionally, and maybe unexpectedly, across generations most respondents felt that they can keep up with technology.

While preferences for new technologies may be limited because of few individuals having experienced using them, it is surprising that none of the generations participating in our study acknowledged the advantages of established welfare technologies and smart homes. In fact, welfare technologies (for example, PERS) have been available and used in healthcare and social services for older adults since the 1970s, and smart home solutions have been available and described at least since the 1980s^24^. Thus, as all three generations have experienced the development of such technologies, this aspect of the results is somewhat surprising. According to the UTAUT2, adoption of technology is based on user characteristics, intention to use and usage behavior^12^. Hence it is likely that respondents base their choices on what they have previous experience with and technologies that they know what to expect of. The preferences for traditional technologies revealed by the present study reflect age-stereotypic attitudes in line with previous findings suggesting that older adults are less interested in technology development and less likely to adopt new technologies^25^. However, other aspects of the results show that attitudes toward technology are shared among respondents from all generations. This is in line with a recent systematic review which identified that neither age nor sex were significantly associated with attitudes toward, for example, social robots^26^. Furthermore, given the ubiquitous deployment of technology, researchers conjecture that age disparities may diminish over time, especially if older adults have access to technology and if designers consider older adults as active users^17^. This more nuanced and complex picture not least identified from the present study should be shared with audiences such as policymakers, public authority officials and the general population to counteract ageist views.

Like the qualitative study^23^ preceding the present survey study in the GenerationTech project, some attitudes toward technologies differed within generations and perspectives were sometimes shared across generations rather than within. Regardless of which generation, respondents considered household devices as practical and necessary to meet their needs, while not always useful or user-friendly. In agreement with recent research^27^ and a Swedish national report^28^, respondents largely wanted to use technologies to remain independent and stay in contact with friends and family. Likewise, all generations shared opinions on home entertainment and exercise and assistive devices as means to support active and healthy aging.

Contrasting the unanimous aspects of the results, the present study displays generational variation in attitudes toward technology and reasons for using technology. For example, the oldest rather than the younger generations reflected on environmental sustainability before adopting new technologies. While this is a finding in line with our qualitative study from the same project^23^, other studies describe climate anxiety to be more prevalent in younger generations^29^. Furthermore, the oldest generation was least willing to use digital technologies or artificial intelligence, and the youngest generation was least likely (and the middle-aged generation most likely) to define social media as a means for active and healthy aging. Previously older adults have been shown to be the ones least likely to adopt social media^15^. Perceived value plays a vital role in the adoption of technology especially among older adults, even more so than previous use of a technology^21^. Furthermore, Lee et al.^17^ speculated that older adults’ attitudes toward technology tend to be more positive. Maybe the result of our study is the first indication of a shift in terms of perceived value, showing that the middle-aged and oldest generations are most likely to adopt social media to support their active and healthy aging. Particularly the younger and middle-aged generations shared opinions concerning ICT and household devices as being useful and saving them time. Different generations of people belong to different technological generations because they experienced different technologies and technology developments during their lifetime, which is a period effect that might explain these findings^16,18^.

According to domestication theory^30^, adaptation to technology is an ongoing process. That is, technology is first integrated into everyday life, which leads to individual adjustment and adaptation to the technology; in turn, it helps the industry innovate new ways for the forthcoming generations. All generations have experiences of technology but in slightly different ways^18,30^, which may unite or divide generations. Further research with a domestication approach could help explain adoption and acceptance using a broader perspective in all generations.

Technology period effects may also explain why the oldest generation perceived technology as less useful and user-friendly, and why their reasons for using technology overall differed from those reported by the younger generations in our study. The perceived usefulness, ease of use, and meaningfulness of technology explains attitudes and acceptance of technology among older adults^22^. In line with the UTAUT^11^, to adopt technology it must meet our expectations, in this case, effort and performance expectancy. If ICT products, for example, for shopping and controlling home entertainment, do not save time or are not designed to target older adults’ needs, they are found less relevant and meaningful to use. In addition, stigmatization describing older adults as less willing to use technology may spur technology developments that target younger user groups^20^ and limit user-friendliness across generations. Overall, as the oldest generation was willing to adopt new technologies, our findings support that this is a technology problem rather than an age problem, thus speaking to the need for more and earlier user involvement in the technology development processes. That is, generational preferences such as the oldest generation’s wish for standard rather than complex and extra functions in digital devices should be taken seriously when developing new user-friendly technologies.

In contrast to existing research, we found that across all three generations represented in our study, but more pronounced in the youngest generation, respondents were able to keep up with technology. That is, previous studies often focused merely on the current generation of older adults and displayed their difficulties in keeping up with technology^19^. Supported by our findings, older people are interested in and want to use technology^31^, and they are familiar with technology and technology development. It may be that experience with technology is resulting in greater comfort and belief in technology, which in turn leads to a perception of being able to use the technology^17^. However, it is important to keep in mind that our study was conducted in Sweden, where Internet access among older people has never been higher^28^. There are cross-national differences in this respect and more research is warranted to shed light on such developments. Based on these first descriptive results from the GenerationTech project, in forthcoming studies results from more complex research questions building on the current results will be reported.

All three generations identified safety as an important reason to use technologies for active and healthy aging; however, in contrast to what could be expected, the oldest generation was least likely to do so. This is interesting because many technologies (for example, PERS and night cameras) are designed and implemented to support safety in later life; other studies showed that older people use smartphone technology to make them feel safe, for example, during out-of-home walks^32^.

Moreover, the oldest generation was least likely to prefer monitoring of health and activity or using Internet-based shopping and Internet services to support active and healthy aging. However, data collection was done before the coronavirus disease 2019 pandemic; with the increased use of digital shopping during the pandemic^28^, preferences may have changed and even caused increased digital inequalities^33^. Up-to-date Swedish data on people born in the 1940s (equivalent to the oldest generation in the present study) show that 38% need help with digital technologies, 7% have no knowledge about the Internet and 17% do not use it^28^. However, because socioeconomic factors such as finances, education, experience and previous exposure to technology in working life have an important role for digital literacy and the possibility to invest in technology^32^, it should be kept in mind that in all generations digital and social exclusion is prevalent.

### Strengths and limitations

While we used a large sampling frame and sent several reminders, the response rate was low, especially in the younger generations. Unfortunately, this limits the generalizability of the results. It is a strength, however, that the sample resembles the Swedish population. For example, 78% of respondents had at least a high school qualification, compared to 85% in the Swedish general population. While 49% of our respondents had a university degree, 42% of Swedish citizens hold such a degree. Like the Swedish general population overall, most respondents were born in Sweden (90%) and lived in a larger or major city (approximately equal to 75%). Respondents represented different socioeconomic classes and nationalities, making the survey sample heterogeneous. Accordingly, the results are of general relevance but should be interpreted with this and risk of bias in mind.

Another noteworthy limitation is that questions about the respondents’ actual use and previous experience with technology and knowledge about and experience with smart home technologies could have been included. However, designing the present survey needed a delicate balance between comprehensiveness and an acceptable respondent burden. In hindsight, including such questions would have made it easier to draw conclusions about how knowledgeable respondents were about the questions they answered. Another possible bias was induced by the eventuality that those who respond to a Web survey have more interest in technology than those who do not respond, affecting the validity of the results regarding the overall perception of technology in our study. That is, although alternative administration modes were offered almost all (95%) responded online. The fact that 98% of Swedish households had access to the Internet at the time of data collection^28^ probably means that this had a minor influence on the results. Finally, the challenge to predict what younger generations will want to support active and healthy aging in a 50-year perspective is an overall potential limitation. Circumstances affect one’s needs and circumstances will undoubtedly change significantly as one ages.

## Conclusion

Research applying a generational perspective to understand acceptance and adoption of different types of technology throughout the aging process was a previously largely unexplored avenue. Unlike previous studies on aging and technology that only focused on older adults, our study captured generational perspectives rarely displayed in research. Through this design, we were able to identify generational differences and similarities that without generational comparison would have been interpreted as attributed to a single age group. This is important because the results show that attitudes toward and acceptance of technologies for active and healthy aging are similar across generations in many ways. Notable differences were nevertheless displayed and deserve attention when developing new technology. Overall our findings are important to consider when developing and implementing technologies for active and healthy aging.

## Methods

### Design

This study was based on a quantitative, cross-sectional survey, part of the GenerationTech project, which is a 3-year research endeavor at Lund University, Sweden. The Swedish Ethical Review Authority (ref. no. 2019-02072) approved the study. Kantar Sifo, a company with documented experience from large-scale data collection, performed the sampling, recruitment and data collection for the present study on behalf of and in collaboration with the research team.

### Respondents, sampling and recruitment

A random sample was drawn from the Swedish State Personal Address Register (SPAR), representing men and women stratified in three age cohorts (30–39, 50–59 and 70–79 years). Based on 2016 population statistics (Statistics Sweden), the 30–39-year-old national cohort was approximately 1.25 million (48.7% women), the 50–59-year-old cohort was approximately 1.25 million (49.3% women) and the 70–79-year-old cohort was approximately 900,000 (51.8% women). We calculated a total sample size of 3,598 to generate estimates with a confidence level of 95% and a margin of error of 4%. To generate this sample, Kantar Sifo acquired 10,000 addresses from SPAR in August 2019. SPAR includes all persons registered as residents in Sweden and is updated each day with data from the Swedish Population Register. Different numbers of addresses were included for the different age cohorts to compensate for the fact that younger individuals have a lower response rate according to Kantar Sifo’s current data collection experiences (Table 1). We planned to continue recruitment until 600 men and 600 women from each age cohort, that is, each stratum, had responded to the survey.

Kantar Sifo first contacted potential respondents by mail, including information for informed consent in line with research ethics, a survey Web link and unique individual login information. Nonresponders were sent a postal reminder after 1 week including the same content as the first letter. Trained staff from Kantar Sifo made up to eight attempts by phone to reach persons who had not responded after 2 weeks, to remind them about the online survey. During the same call, respondents could respond to the survey via a telephone interview, on verbal informed consent, or get a postal version of the survey sent by mail to their home address. Potential respondents, who said they would respond but did not within 2–3 weeks, received an additional reminder replicating the original information once more. A synchronized system was used by Kantar Sifo to safeguard that no responders received a reminder. These combined efforts resulted in a final sample of 2,121 respondents including 1,081 (51%) men (response rate 22%) and 1,040 (49%) women (response rate 21%), divided into three generation cohorts (30–39, 50–59 and 70–79). The youngest generation included 639 respondents (49% men and 51% women). The middle-aged generation included 703 respondents, with 49% men and 51% women. The oldest generation included 779 respondents with 54% men and 46% women (Table 1). All respondents gave written informed consent before starting the survey.

### Data collection

Data were collected through a questionnaire developed for GenerationTech based on qualitative findings^23^ involving the same age cohorts as the present study from the same project and from relevant scientific literature. The survey included 24 questions on attitudes to, and acceptance of, a broad range of technology, including products and services used in everyday activities (for example, household devices, kitchenware, cars, new lightbulbs, TVs), ICT (for example, smartphones, tablet computers, computers), welfare technology (for example, safety alarms, video surveillance, e-health) and medical technology (for example, assistive technology such as wheeled walkers, wheelchairs and communication aids and medical products such as pacemakers or insulin pumps). The questionnaire also included seven questions about respondent characteristics such as education, occupation, housing, civil state and country of birth, as well as self-rated general health, life satisfaction and finances to cover technology needs. The estimated time required to complete the survey was 10–15 min.

A pilot study was conducted with 21 men and women representing the three age cohorts recruited via the Kantar Sifo Web panel. The Kantar Sifo Web panel includes a representative sample of the Internet-using general population in Sweden and was considered relevant for the pilot study. The results of the pilot required only a few changes to the survey (for example, one response alternative was removed because the pilot respondents did not find it relevant and the ‘Other’ response alternative at the end of most questions was rephrased).

During the data collection, Kantar Sifo performed regular quality control of the data, focusing on correct, complete and logical recording in the database, and communicated with the GenerationTech research team when needed. Researchers monitored data collection to identify potential systematic errors when 10% of the data were collected and listened to 5% of the phone interviews to ensure quality. The researchers and Kantar Sifo also engaged in active dialog during the process to ensure that processes were followed as intended.

### Statistics and reproducibility

A national cross-sectional survey was conducted with randomly sampled men and women from three different generations (30–39, 50–59 and 70–79-year-olds). To predetermine sample size, we calculated a total sample size of 3,598 to generate estimates with a confidence level of 95% and a margin of error of 4%. To generate this sample, 10,000 addresses were randomly drawn from SPAR. The final sample of 2,121 men and women was divided into three different generations. The youngest generation included 639 respondents. The middle-aged generation included 703 respondents. The oldest generation included 779 respondents. Data collection and analysis were not performed blind to the conditions of the experiments. Descriptive statistics were used to describe the three-generation sample with regard to basic demographics. Differences between generations regarding products and characteristics preferences were investigated using chi-squared tests. Data were not normally distributed and met the assumptions of the statistical tests used. The alpha level was set to *P* < 0.05 and Bonferroni-corrected. Binary logistic regression was implemented to investigate differences between generations in terms of attitudes toward household devices and ICT products, respectively. The dependent variable was attitude toward household devices or ICT and the independent variable was which generation they belonged to. To control for socio-demographic characteristics, country of birth, education, size of municipality, self-rated economy, life satisfaction and general health were entered as confounders in an adjusted model. No data were excluded from the analyses. SPSS v.27 (IBM Corporation) was used for the data analyses.

### Reporting summary

Further information on research design is available in the Nature Portfolio Reporting Summary linked to this article.

### Supplementary information

Reporting Summary

## Figures and Tables

**Extended Data Table 1:**
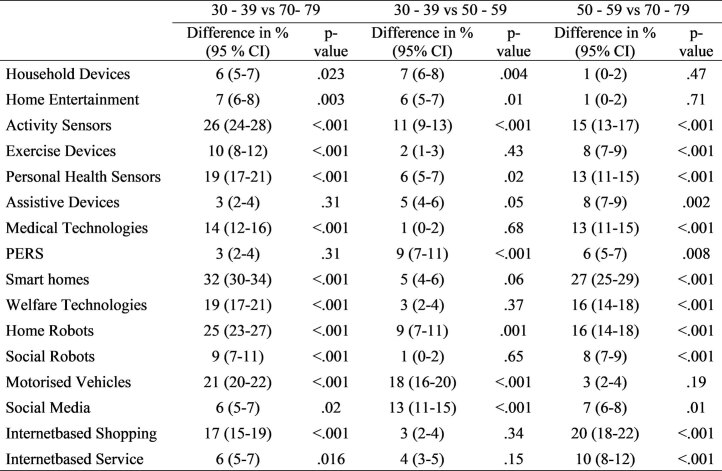
Differences between generations in terms of what kind of technologies respondents want to use to support active and healthy aging (*n* = 2,121)

All pairwise comparisons were analyzed with a two-sided chi-squared test (*P* < 0.05). All *P* values were Bonferroni-corrected.

**Extended Data Table 2:**
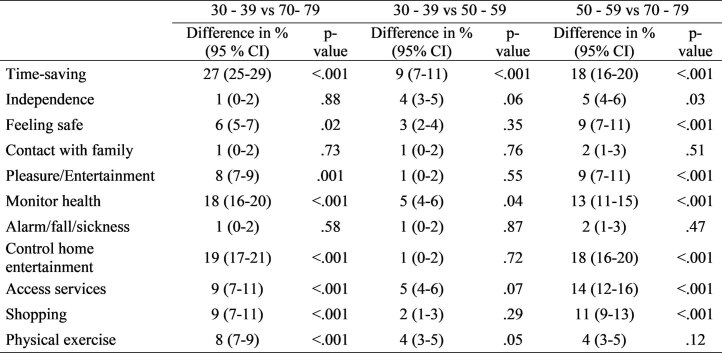
Differences between generations in terms of reasons for using technologies to support active and healthy aging (*n* = 2,121)

All pairwise comparisons were analyzed with a two-sided chi-squared test (*P* < 0.05). All *P* values were Bonferroni-corrected.

**Extended Data Table 3:**
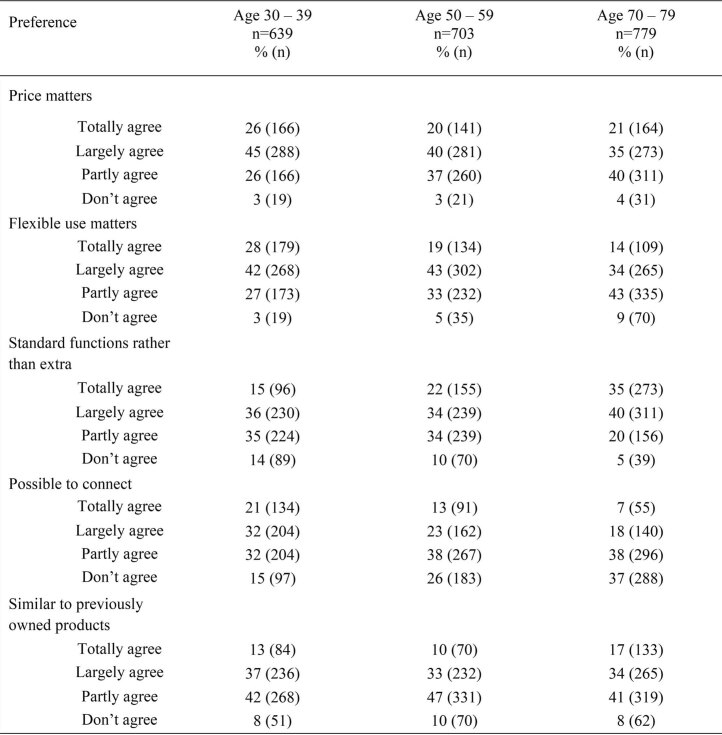
Respondents’ preferences, within each generation, when choosing new technology (*n* = 2,121)

Percentages rounded to the nearest integer.

**Extended Data Table 4:**
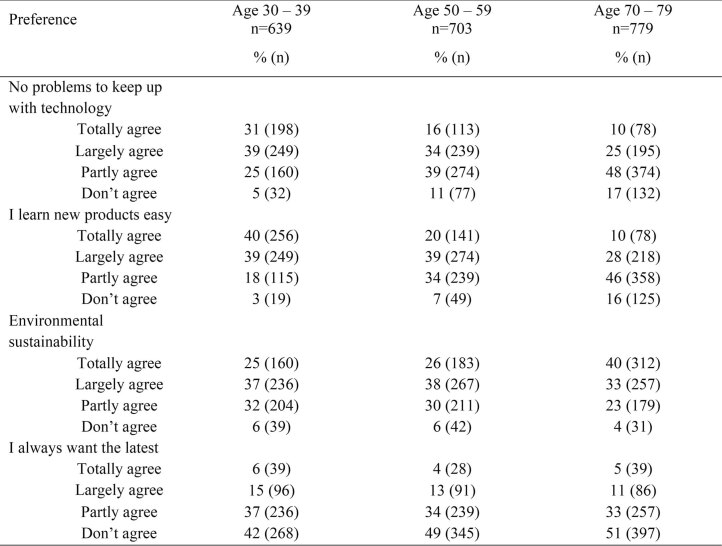
Respondents’ preferences, within each generation, when adopting new technology (*n* = 2,121)

Percentages were rounded to the nearest integer.

## Data Availability

The data used in this study contain sensitive information about the study participants who did not provide consent for public data sharing. The current approval by the Swedish Ethical Review Authority (ref. no. 2019-02072) does not include data sharing. A minimal dataset containing anonymous data used in the present study could be shared if requested by a qualified academic investigator for the sole purpose of replicating the present study, provided that data transfer is in agreement with European Union legislation on general data protection regulation and approval by the Swedish Ethical Review Authority (Department of Health Sciences, Lund University, DHSdataaccess@med.lu.se). The principal investigator was S. Iwarsson (susanne.iwarsson@med.lu.se).
