# Supplementary material for: Attitudes related to technology for active and healthy aging in a national multigenerational survey
Source: Nat Aging. 2023 Apr 6;3(5):617–25. doi: 10.1038/s43587-023-00392-3 (PMC10191841; doi:10.1038/s43587-023-00392-3)
Supplement: Supplementary file 1 — Reporting Summary [file 43587_2023_392_MOESM1_ESM.pdf]

nature portfolio

Corresponding author(s): Jens Offerman

Last updated by author(s): Jan 30, 2023

Reporting Summary

Nature Portfolio wishes to improve the reproducibility of the work that we publish. This form provides structure for consistency and transparency in reporting. For further information on Nature Portfolio policies, see our Editorial Policies and the Editorial Policy Checklist.

Statistics

For all statistical analyses, confirm that the following items are present in the figure legend, table legend, main text, or Methods section.

n/a	Confirmed	
☐	☒	The exact sample size (*n*) for each experimental group/condition, given as a discrete number and unit of measurement
☐	☒	A statement on whether measurements were taken from distinct samples or whether the same sample was measured repeatedly
☐	☒	The statistical test(s) used AND whether they are one- or two-sided<br>*Only common tests should be described solely by name; describe more complex techniques in the Methods section.*
☐	☒	A description of all covariates tested
☒	☐	A description of any assumptions or corrections, such as tests of normality and adjustment for multiple comparisons
☐	☒	A full description of the statistical parameters including central tendency (e.g. means) or other basic estimates (e.g. regression coefficient) AND variation (e.g. standard deviation) or associated estimates of uncertainty (e.g. confidence intervals)
☐	☒	For null hypothesis testing, the test statistic (e.g. *F*, *t*, *r*) with confidence intervals, effect sizes, degrees of freedom and *P* value noted<br>*Give P values as exact values whenever suitable.*
☒	☐	For Bayesian analysis, information on the choice of priors and Markov chain Monte Carlo settings
☒	☐	For hierarchical and complex designs, identification of the appropriate level for tests and full reporting of outcomes
☒	☐	Estimates of effect sizes (e.g. Cohen's *d*, Pearson's *r*), indicating how they were calculated

Our web collection on statistics for biologists contains articles on many of the points above.

Software and code

Policy information about availability of computer code

Data collection	No software was used, however a representative sample was drawn
Data analysis	IBM SPSS Statistics 27 was used for the data analyses.

For manuscripts utilizing custom algorithms or software that are central to the research but not yet described in published literature, software must be made available to editors and reviewers. We strongly encourage code deposition in a community repository (e.g. GitHub). See the Nature Portfolio guidelines for submitting code & software for further information.

Data

Policy information about availability of data

All manuscripts must include a data availability statement. This statement should provide the following information, where applicable:

- Accession codes, unique identifiers, or web links for publicly available datasets
- A description of any restrictions on data availability
- For clinical datasets or third party data, please ensure that the statement adheres to our policy

The data used in this study contains sensitive information about the study participants and they did not provide consent for public data sharing. The current approval by the Regional Ethical Review Board in Lund, Sweden (No. DBPR) does not include data sharing. A minimal data set could be shared by request from a qualified academic investigator for the sole purpose of replicating the present study, provided the data transfer is in agreement with EU legislation on the general data protection regulation and approval by the Swedish Ethical Review Authority. Contact information: Department of Health Sciences, Lund University Box 157, 221 00 Lund, Sweden. Contact address: DHSdataaccess@med.lu.se Principal investigator: Professor Susanne Iwarsson susanne.iwarsson@med.lu.se Swedish Ethical Review Authority, Box 2110, 75 002 Uppsala, Sweden. Phone: +46 10 475 08 00.

Field-specific reporting

Please select the one below that is the best fit for your research. If you are not sure, read the appropriate sections before making your selection.

☐ Life sciences ☒ Behavioural & social sciences ☐ Ecological, evolutionary & environmental sciences

For a reference copy of the document with all sections, see nature.com/documents/nr-reporting-summary-flat.pdf

Behavioural & social sciences study design

All studies must disclose on these points even when the disclosure is negative.

Study description	Quantitative cross-sectional study design, a national survey
Research sample	A random sample of men and women from Sweden divided into three different age cohorts (30-39, 50-59 and 70-79). Demographic information about participants includes age, gender, self-rated health, life satisfaction and economy, housing, civil state, occupation and where they live. The sample resembles the Swedish population making it a representative sample.
Sampling strategy	A random sample was drawn from the Swedish State Personal Address Register (SPAR), representing men and women stratified in three age cohorts (30-39, 50-59 and 70-79 years-old).<br>To ensure an equal number of respondents in each age cohort. Different numbers of addresses were included for the different age cohorts to compensate for the fact that younger persons have a lower response rate according to KS's current data collection experiences. We calculated a total sample size of 3,598 to generate estimates with a confidence level of 95% and a margin of error of 4 (Cochran 1977).
Data collection	Kantar Sifo first contacted potential respondents through postal letters, including information for informed consent in line with research ethical principles, a survey web-link, and unique individual login-information. Non-responders were sent a postal reminder after one week including the same content as the first letter. Trained staff from Kantar Sifo made up to eight attempts by phone to reach persons who had not responded after two weeks, to remind about the online survey. The Kantar Sifo staff was blinded to the study hypothesis. Most participants responded online (n=2015) without any researcher present. During telephone interviews only one staff member from Kantar Sifo and the respective participant were present. However, one of the researchers (Co-author SF) listened in to 5% of the interviews to secure the data collection quality.
Timing	Data were collected between the 20th of August 2019 and the 4th of November 2019.
Data exclusions	No data were excluded
Non-participation	2879 out of 5000 drawn samples declined to answer the questionnaire, leading to a final sample of 2121 participants.
Randomization	Participants were allocated to one of three different groups based on their age. The three groups were 30 - 39 age cohort, 50 - 59 age cohort and 70 - 79 age cohort.

Reporting for specific materials, systems and methods

We require information from authors about some types of materials, experimental systems and methods used in many studies. Here, indicate whether each material, system or method listed is relevant to your study. If you are not sure if a list item applies to your research, read the appropriate section before selecting a response.

Materials & experimental systems

n/a	Involved in the study	
☒	☐	Antibodies
☒	☐	Eukaryotic cell lines
☒	☐	Palaeontology and archaeology
☒	☐	Animals and other organisms
☐	☒	Human research participants
☒	☐	Clinical data
☒	☐	Dual use research of concern

Methods

n/a	Involved in the study	
☒	☐	ChIP-seq
☒	☐	Flow cytometry
☒	☐	MRI-based neuroimaging

Human research participants

Policy information about studies involving human research participants

Population characteristics	Three age cohorts (30-39, 50-59 and 70-79) of men and women (n=2121)

Recruitment	Kantar Sifo, Sweden recruited all participants for this study. Participants were randomly drawn from the Swedish State Personal Address Register (SPAR).
Ethics oversight	The Swedish Ethical Review Authority

Note that full information on the approval of the study protocol must also be provided in the manuscript.
